# Correction: Nano *N*-TiO_2_ mediated selective photocatalytic synthesis of quinaldines from nitrobenzenes

**DOI:** 10.1039/d0ra90111c

**Published:** 2020-10-20

**Authors:** Kaliyamoorthy Selvam, Meenakshisundaram Swaminathan

**Affiliations:** Department of Chemistry, Annamalai University Annamalainagar 608 002 India chemres50@gmail.com +91 4144 225072

## Abstract

Correction for ‘Nano *N*-TiO_2_ mediated selective photocatalytic synthesis of quinaldines from nitrobenzenes’ by Kaliyamoorthy Selvam *et al.*, *RSC Adv.*, 2012, **2**, 2848–2855, DOI: 10.1039/C2RA01178F.

The authors regret omitting citations of their related papers in *Journal of Molecular Catalysis A: Chemical* and *Applied Catalysis A: General*: ‘Cost effective one-pot photocatalytic synthesis of quinaldines from nitroarenes by silver loaded TiO_2_’ (DOI: 10.1016/j.molcata.2011.09.014)^1^ and ‘Mesoporous nitrogen doped nano titania—A green photocatalyst for the effective reductive cleavage of azoxybenzenes to amines or 2-phenyl indazoles in methanol’ (DOI: 10.1016/j.apcata.2011.11.011).^2^ The citations should have appeared in the following places as ref. 36 (ref. 1, in the reference list here) and ref. 37 (ref. 2, in the reference list here):

In the sentence starting on line 5 of paragraph 5 in the introduction:

‘Photocatalytic synthesis of quinolone derivatives from nitrobenzene using TiO_2_, metal doped TiO_2_ and others had been reported earlier.^1^^,23–25^’

At the end of Section 3.12 with the addition of the following sentence:

‘This catalyst was also found to be effective for the reductive cleavage of azoxybenzenes to amines or 2-phenyl indazoles in methanol.^2^’

The authors regret that it was not clear in the original article that the bare TiO_2_ and *N*-TiO_2_ characterisation data had been reproduced from their related *Journal of Molecular Catalysis A: Chemical*, *Applied Catalysis A: General* and *Catalysis Communications* papers.^1–3^ Although the *Catalysis Communications* article was cited as ref. 25 (ref. 3, in the reference list here) in the original article, it was not made clear that some of the data was reproduced from this article. The appropriate figure captions have been updated to reflect this.

Fig. 2: Diffuse reflectance spectra of (a) bare TiO_2_, (b) *N*-TiO_2_ and (c) TiO_2_-P25. The bare TiO_2_ data in Fig. 2a have been reproduced with permission from ref. 1. Copyright 2011 Elsevier. The *N*-TiO_2_ data in Fig. 2b have been reproduced with permission from ref. 2. Copyright 2012 Elsevier.

Fig. 3: Photoluminescence spectra of (a) bare TiO_2_, (b) TiO_2_-P25 and (c) *N*-TiO_2_. The bare TiO_2_ data in Fig. 3a have been reproduced with permission from ref. 1. Copyright 2011 Elsevier. The *N*-TiO_2_ data in Fig. 3c have been reproduced with permission from ref. 2. Copyright 2012 Elsevier.

Fig. 4: HR-TEM analysis: (a and b) images at two different regions of *N*-TiO_2_, (c) SAED pattern of *N*-TiO_2_, (d) lattice fringes of *N*-TiO_2_ and (e) particle size distribution of *N*-TiO_2_. Fig. 4 has been entirely reproduced with permission from ref. 2. Copyright 2012 Elsevier.

Fig. 5: X-ray photoelectron spectra of *N*-TiO_2_: (a) survey spectrum, (b) Ti 2p peak, (c) O 1s peak, (d) N 1s peak and (e) C peak. Fig. 5 has been entirely reproduced with permission from ref. 2. Copyright 2012 Elsevier.

Fig. 6: (a) N_2_ adsorption–desorption isotherms of *N*-TiO_2_ and (b) its pore size distribution. Fig. 6 has been entirely reproduced with permission from ref. 2. Copyright 2012 Elsevier.

Fig. 8: GC-MS chromatograms at different reaction times for the photocatalytic conversion of nitrobenzene with *N*-TiO_2_. Fig. 8 has been entirely reproduced with permission from ref. 3. Copyright 2011 Elsevier.

The authors also wish to remove Fig. 1 from the original article due to similarities between two of the spectra and the raw data no longer being available. This does not affect the conclusions as the presence of nitrogen was confirmed by other techniques.

The authors also wish to clarify the differences between this *RSC Advances* paper and the *Journal of Molecular Catalysis A: Chemical*, *Applied Catalysis A: General* and *Catalysis Communications* papers.^1–3^ The *Journal of Molecular Catalysis A: Chemical* paper discusses the photocatalytic synthesis of quinaldines from nitroarenes by silver loaded TiO_2_.^1^ The *Applied Catalysis A: General* paper reports the reductive cleavage of azoxybenzenes to amines or 2-phenyl indazoles using mesoporous nitrogen doped nano titania.^2^ The *Catalysis Communications* paper, ref. 25 in the original article, discusses the synthesis of quinaldines from nitroarenes with gold loaded TiO_2_ nanoparticles.^3^ The original *RSC Advances* paper discusses the catalytic ability of *N*-TiO_2_ in the synthesis of quinaldines from nitrobenzenes. In each paper, either a different catalyst was used or a different synthetic reaction was investigated.

## Supplementary Material
